# Micronutrients and Pregnancy

**DOI:** 10.3390/nu14030585

**Published:** 2022-01-28

**Authors:** Faruk Ahmed

**Affiliations:** Public Health, School of Medicine and Dentistry, Gold Coast Campus, Griffith University, Gold Coast, QLD 4220, Australia; f.ahmed@griffith.edu.au

Micronutrient deficiencies are known to affect more than two billion people globally [1]. The magnitude of the problem is much greater in low-income countries, where multiple micronutrient deficiencies often occur concurrently as a result of poor-quality diets [2]. Suboptimal dietary intake of micronutrients during pregnancy has been associated with low micronutrient status, poor foetal growth, preterm delivery, poor infant survival, and increased risk of chronic diseases in later life [1,3,4]. Hence, adequate micronutrient status and good dietary practice during pregnancy are necessary to achieve healthy birth outcomes. This Special Issue on “Micronutrients and Pregnancy” has collated eight manuscripts, of which four are original articles and four are reviews.

Several articles in this issue focus on anaemia and iron deficiency. In a systematic review including 44 studies published from 1975 to January 2021, Quezada-Pinedo et al. [5] reported on maternal iron status in pregnancy and its association with a wide range of health outcomes in the infants after birth. In addition, the authors conducted meta-analyses of 16 studies of biochemical outcomes; they reported a significant association between maternal and child iron status based on soluble transferrin receptor concentrations, a reliable biomarker of low iron status, which is affected to a lesser degree by inflammation. Twelve out of twenty studies that examined neurodevelopment (cognition, motor function, language, and memory) found better outcomes in children of mothers with higher iron status, although in one study, harmful effects from higher iron status was reported. It is important to note that 73% (*n* = 32) of the studies in this systemic review measured child health outcomes in early infancy while the remainder measured outcomes in children whose ages ranged from 24 h after birth until school age. Quezada-Pinedo et al. concluded that there was a need for more high-quality studies examining the long-term effects of maternal iron status during pregnancy on child health outcomes.

In a narrative review including 14 articles published in the 10 years up until 2021, Wawer et al. [6] reported that pre-pregnancy obesity and/or overweight or obesity during pregnancy posed a significantly greater risk of iron deficiency/iron deficiency anaemia (ID/IDA) for women and their babies both during pregnancy and postpartum. Based on the available literature, the authors also reported that the increased risk of ID/IDA in overweight or obese pregnant women could be due to the metabolic effects of inflammatory responses, complications, and a higher rate of obstetric interventions that could lead to greater blood loss. Wawer et al. suggested introducing monitoring of trimester-specific iron and inflammatory status of overweight or obese pregnant women during antenatal and postnatal care so that tailored treatment could be provided to reduce the risk of ID/IDA in pregnancy and the postpartum period, thereby potentially reducing the risk of adverse pregnancy outcomes.

The World Health Organization recommends iron and folic acid (IFA) supplementation for all pregnant women to prevent anaemia and adverse foetal outcomes [7]. In a study from the Philippines, Felipe-Dimog et al. [8] used data from the 2017 Philippine National Demographic and Health Survey to report on compliance with the recommended IFA supplementation in pregnant women. They also reported on the factors associated with poor compliance. Overall, three in four women did not comply with the IFA supplementation recommendation. Pregnant women of Islamic faith and non-Indigenous Muslim ethnicity were found to be less likely to comply with the recommended IFA supplementation. On the other hand, women aged between 25 and 34 years, those with better education and higher wealth status, rural residents, and those who visited antenatal clinics during the first trimester of pregnancy and at least four times during their pregnancy were significantly positively associated with compliance with IFA supplementation. Felipe-Dimog et al. concluded that there was a need to develop strategies targeted at specific groups such as religious minorities, poor urban residents, the less educated, and young women to motivate early and regular antenatal care visits and improve compliance with IFA supplementation recommendations.

The original research by Ahmed et al. [9] found a prevalence of vitamin D insufficiency and deficiency among pregnant women from rural Bangladesh. Overall, there was a high prevalence of hypovitaminosis D in this population group, with 17.3% having vitamin D deficiency (serum 25(OH)D concentration < 30.0 nmol/L) and with 47.2% having vitamin D insufficiency (serum 25(OH)D concentration between 30–50 nmol/L). The authors also examined the potential risk factors of vitamin D deficiency and insufficiency in this population group. Parity (nullipara) and gestational age (first trimester) appeared to be the common risk factors of vitamin D deficiency and insufficiency. A husband’s occupation, and anaemia were found to be important predictors of vitamin D insufficiency, whereas being of younger age and suboptimal vitamin A status appeared to be the risk factors of vitamin D deficiency [9]. While a major limitation of this study was the use of convenience sampling for selecting the study participants, therefore introducing potential bias, the authors concluded that hypovitaminosis D was common among pregnant women in rural Bangladesh and recommended comprehensive intervention strategies including low-dose vitamin D supplementation.

Using data from a large Norwegian pregnancy cohort (*n* = 71,728), Solé-Navais et al. [10] reported an association between selenium intake from the diet during the first half of pregnancy and increased birth weight and decreased risk of small for gestational age (SGA) status, after adjusting for various confounders. However, the authors did not find such an association for organic and inorganic selenium intake from supplements. The authors indicated that one of the possible reasons for the lack of association could be the difficulty in accurately measuring the intake of selenium from the supplements. Furthermore, on a subsample analysis with available blood selenium (*n* = 2628), the authors failed to observe any association between whole blood selenium concentration and birth weight or SGA. Solé-Navais et al. pointed out that this lack of association could be due to the homogenous group of subsamples with fairly good selenium status. The authors concluded that, while a maternal diet rich in selenium during pregnancy may be beneficial for foetal growth, further interventional studies were needed to confirm the causal relationship.

The in vitro study by Habibi et al. [11] examined the protective effects of different doses of selenium, iodine, and copper on the first-trimester human placenta against oxidative stress response. The authors found a significant reduction in DNA damage and apoptosis when placenta explants were treated with sodium selenite and potassium iodide. Following the induction of oxidative stress, a supraphysiological concentration of sodium selenite (1.6 µM) and both concentrations of potassium iodide (0.5 and 1 µM) were found to result in significant reductions in DNA damage and apoptosis, while increased apoptosis and DNA damage were observed when treated with a high concentration of copper (40 µM). However, the effect was no longer significant after the induction of oxidative stress. The authors concluded that an optimal level of micronutrients may help to ensure healthy placental development.

In a systematic review including 12 articles and a series of publications (4 articles) published prior to October 2020, Zgliczynska and Kosinska-Kaczynska [12] reported on the supply and needs of mothers with multifetal pregnancy regarding micronutrients and the epidemiology of deficiencies in this population. As indicated by the authors, this review suffered from some limitations, which included a small sample size, a lack of randomized sampling, and an interventional study. Based on the available studies, the authors concluded that women with multifetal pregnancies might be at risk of vitamin D and iron deficiencies. The evidence for other microelements is either inconsistent, scarce, or absent. Zgliczynska and Kosinska-Kaczynska emphasized the need for more in-depth prospective and population studies to ascertain if nutritional recommendations for pregnant women required adjustments in cases of multifetal pregnancy.

In another review including 85 studies published between 1981 and 2020, Jouanne et al. [13] reported on the current recommendations in the United States, Canada, and Europe for the dietary intake of various micronutrients and omega-3 fatty acids during pregnancy. They also discussed the potential benefits of each form of supplementation. Jouanne et al. concluded that the current recommended daily intakes seemed to overestimate the actual needs of pregnant women. According to the authors, for well-nourished, healthy women who have access to a varied diet to carry a normal pregnancy to term, there was no need for any supplementation except that provided by their food consumption during pregnancy. However, the authors also recommended a personalized approach to nutritional counselling that takes into account a woman’s body mass index, socioeconomic status, access to food, race, ethnicity, and cultural food choices.

The collection of articles in this issue brings together the latest research on a broad range of topics on micronutrients and pregnancy, including the prevalence of various micronutrient deficiencies and their risk factors, as well as highlights the potential benefits of adequate micronutrient and/or risk of deficiencies on children’s birth outcomes. I am sure that the findings of these studies will contribute to enhancing our current knowledge of the importance of micronutrients during pregnancy and will encourage readers to conduct further studies in this important area of research.

## Data Availability

Not applicable.
